# The Comparison of Antioxidant Effect of Aspirin, Metformin, Atorvastatin and Captopril Co-administration in the Heart and Kidney Tissues of Diabetic Rats

**DOI:** 10.22037/ijpr.2019.112004.13481

**Published:** 2021

**Authors:** Maryam Paseban, Saeed Niazmand

**Affiliations:** a *Department of Physiology, School of Medicine, Mashhad University of Medical Sciences, Mashhad, Iran. *; b *Applied Biomedical Research Center, Mashhad University of Medical Sciences, Mashhad, Iran.*

**Keywords:** Diabetes, Metformin, Captopril, Atorvastatin, Aspirin, Heart, Kidney

## Abstract

The present study investigated the effects of co-administration of aspirin, metformin, atorvastatin and captopril on serum lipid profile and oxidative stress in the heart and kidney of streptozotocin-induced diabetic rats. In this study, rats were randomly divided into the following eleven groups: control (Cont.), and diabetic (D), as well as 9 groups that were treated with metformin (M, 300 mg/kg) or aspirin (ASA, 120 mg/kg) alone or in different combinations with captopril (C, 50 mg/kg), or atorvastatin (AT, 40 mg/kg), as follows: (D + M), (D + ASA), (D + M + ASA), (D + M + C), (D + M + AT), (D + M + C + ASA), (D + M + C + AT), (D + M + AT + ASA), and (D + M + C + AT + ASA). The rats in treatment groups daily received drugs by gavage for six weeks. Finally, serum lipid profile and levels of oxidative markers in the heart and kidney tissues were evaluated. In diabetic rats, blood levels of glucose, cholesterol, TG (triglyceride), LDL (low-density lipoprotein), MDA (malondialdehyde) and AIP (atherogenic index of plasma) significantly increased but those of HDL (high-density lipoprotein) and total thiol as well as SOD (superoxide dismutase) and CAT (catalase) activities significantly decreased. Treatment with different combinations of C, ASA, AT and M significantly ameliorated these parameters. This study showed that co-administration of ASA, M, C and AT, could improve glucose and lipid metabolism and oxidative stress markers in the kidneys and heart tissues of diabetic rats more markedly than the administration of these drugs alone.

## Introduction

Diabetes mellitus (DM) is associated with an increased risk of cardiovascular and renal diseases (1, 2). Oxidative stress plays a pivotal role in the development of cardiovascular and renal complications of diabetes. Reactive oxygen species (ROS) were shown to exert a direct inhibitory effect on myocardial function and cause oxidation of various molecules; also, they induce redox genes overexpression, intracellular calcium overload, and DNA fragmentation, which all damage myocardial cells (3). Oxidative stress is also an important pathogenic mechanism underlying chronic kidney disease (4). It is becoming increasingly evident that cellular function changes that may induce oxidative stress play a key role in the development and progression of diabetic nephropathy (DN) (5).

Metformin is an anti-hyperglycemic drug that was shown to improve cardiovascular complications of diabetes (6). Metformin could reduce intracellular ROS levels by increasing the activity of the antioxidative system (7).

The role of the renin-angiotensin-aldosterone system (RAAS) and variations in genes encoding different components of RAAS in susceptibility towards diabetic nephropathy and cardiomyopathy were investigated in numerous studies. The effects of variants of some RAAS constituents genes, including angiotensin-converting enzyme (ACE), as well as angiotensinogen and angiotensin II type 1 receptor (AT1R), on the risk of DN, were extensively studied (8, 9). ACE inhibitors such as captopril were shown to attenuate the progression of diabetes-induced cardiac and renal impairments (10). These agents also protect the cardiovascular system and kidneys against ROS, probably via their superoxide anion scavenging potential (10, 11).

Dyslipidemia is commonly found in diabetic patients (12). Statins such as atorvastatin are used as lipid-lowering drugs in diabetic patients to reduce the risk of cardiovascular diseases (13). Atorvastatin, possibly through boosting antioxidant markers, can preserve myocardial structure which is considered a non-lipid lowering benefit of statins (14). Statins reduce ROS generated by vascular NAD(P)H oxidase and antagonize the pro-oxidant effect of angiotensin II (15). The antioxidant effect of statins contributes to inhibition of myocardial hypertrophy and remodeling and modulation of vascular tone (16). Statins treatment prevented glomerular injury, independent of their cholesterol-lowering effects and it was suggested that such beneficial effect might be mediated through pleiotropic effects including an anti-inflammatory activity by suppression of oxidative stress, NFκB activation, and induction of ICAM-1 expression and macrophage infiltration in the early phase of DN (17). 

Aspirin (ASA) is widely recommended for primary prevention of cardiovascular events in DM (18, 19). In 2013, to prevent cardiovascular diseases, the American Diabetes Association proposed the use of aspirin in diabetic patients with a high risk of cardiovascular complications (20).

Aspirin protects the cardiovascular system by reducing the production of superoxide and preventing the reduction of catalase and superoxide dismutase activity (21). According to the development of diabetes in association with statin use, the introduction of novel therapeutic approaches for the treatment of diabetes is of crucial importance. Recent researches reported that high-dose ASA affects glucose and lipid metabolism (22-24). Also, as high-dose ASA, unlike statins, can inhibit serine kinase IKKβ activity, it might help overcome insulin resistance and improve glucose tolerance in patients with type 2 diabetes (25, 26).

This study was carried out to determine the effect of high-dose ASA alone and in combination with metformin, atorvastatin and captopril on oxidative stress in the heart and kidney tissues of streptozotocin (STZ)-induced diabetic rats.

## Experimental

*Chemicals and drugs*

All drugs were obtained from Sigma (Germany). 

*Animals*

Eighty-eight male Wistar rats (250–280 g and 10 weeks old) were obtained from Central Animal House of Mashhad University of Medical Sciences, Mashhad, Iran. They were kept at 22 ± 1°C with 12 h/12 h light/dark cycles, and they had *ad libitum* access to food and water. All experiments were approved by the Animal Experimentation Ethics Committee of Mashhad University of Medical Sciences (Approval No. 89529). 

*The protocol used to induce diabetes*

Diabetes was induced by a single intraperitoneal (i.p.) dose of streptozotocin (STZ, 60 mg/kg) injected immediately after dissolving the agent in distilled water. Three days after STZ injection, we confirmed the induction of diabetes by measuring fasting glucose levels in blood samples collected after 8-12 hour fasting from orbital sinus. Rats with blood glucose level ≥ 250 mg/dL were considered diabetic (27).

*Experimental design*

Rats were randomly divided into the following eleven groups (n = 8 in each group): control (Cont.), diabetic rats (D), diabetic rats treated with metformin (D + M), diabetic rats treated with aspirin (D + ASA), diabetic rats treated with metformin and aspirin (D + M + ASA), diabetic rats treated with metformin and captopril (D + M +C), diabetic rats treated with metformin and atorvastatin (D + M + AT), diabetic rats treated with metformin, captopril and aspirin (D + M + C + ASA), diabetic rats treated with metformin, captopril and atorvastatin (D + M + C + AT), diabetic rats treated with metformin, atorvastatin and aspirin (D + M + AT + ASA), diabetic rats treated with metformin, captopril, atorvastatin and aspirin (D + M + C + AT + ASA) which were treated (by gavage) daily for six weeks. Normal saline was orally administered (by gavage) to the control and diabetic groups and the other groups received metformin (300 mg/kg), aspirin (120 mg/kg), captopril (50 mg/kg), and atorvastatin (40 mg/kg). All drugs were dissolved in normal saline.

*Preparation and analysis of the samples*

Ether was used to induce anesthesia and the blood samples were obtained from rats orbital sinus. The samples were centrifuged at 3000 rpm and the serum was kept at -20 °C until analysis. Serum fasting glucose concentration was measured at three different time-points namely, before STZ injection (day 0), three days after STZ injection (day 3, when diabetes was confirmed), and six weeks after STZ injection (day 45). Serum cholesterol, triglyceride, LDL, and HDL were measured on day 0 and 45 using Pars Azmoon kits (Iran). The atherogenic index of plasma (AIP) was calculated as log (TG/HDL-C) (28). At the end of the experimental period, rats were sacrificed following induction of deep anesthesia by ether. The heart and kidney tissues were quickly removed and washed with normal cold saline. The heart and kidney samples were then immediately homogenized. Supernatant fractions were obtained by centrifugation of the homogenates at 5000 ×g for 10 min and stored at -80 °C until measurement of oxidative stress parameters levels. 

*Measurement of total thiol*

Total thiol was determined by the method previously described by Sedlak and Lindsay (29). The heart or kidney tissues were homogenized using PBS (phosphate-buffered saline). The supernatant of homogenized heart or kidney (50 μL) was added to 1 mL Tris-EDTA (ethylenediaminetetraacetic acid) buffer (pH 8.6) and the absorbance was read at 412 nm against Tris-EDTA buffer alone (A1). Then, 20 μL of 2, 2’ dinitro 5, 5’ dithiodibenzoic acid (DTNB) (10 mM in methanol) was mixed with the supernatant and the absorbance was read again (A2). The absorbance of the DTNB reagent was also read as blank (B). Total thiol was calculated using the following equation and expressed as mmol/g tissue (30).

Total thiol concentration (mM) = (A2 − A1 − B) × 1.07/(0.05 × 14150)

*Assessment of lipid peroxidation*

Malondialdehyde (MDA) level is an index of lipid peroxidation extent. MDA reacts with thiobarbituric acid (TBA) as a TBA reactive substance (TBARS) and produces a red complex. Briefly, the heart or kidney tissues were homogenized using PBS. Then, 1 ml of the supernatant of homogenized heart or kidney was added to 2 mL of a complex solution containing TBA, trichloroacetic acid (TCA), and hydrochloric acid (HCl); then, the mixture was boiled in a water bath for 40 min. After reaching room temperature, the solution was centrifuged at 1000 ×g for 10 min and the absorbance of the supernatant was read at 535 nm. The MDA levels were expressed as µmol/g tissue weight, using an extinction coefficient of 1.56 × 10^5^ cm^-1^ M^-1 ^(31, 32).

*Determination of superoxide dismutase (SOD) activity*

Superoxide dismutase (SOD) activity was measured by the protocol explained by Madesh and Balasubramanian (33). This colorimetric assay involves the generation of superoxide by pyrogallol auto-oxidation and inhibition of superoxide-dependent reduction of the tetrazolium dye, MTT (3-(4, 5-dimethylthiazol-2-yl) 2, 5-diphenyltetrazolium bromide) to formazan by SOD; the absorption of the solution was measured at 570 nm. One unit of SOD activity was defined as the amount of enzyme causing 50% inhibition in the MTT reduction rate (34).

*Measurement of catalase (CAT) activity*

To determine CAT activity, the method described by Aebi’s was employed (35). This assay is based on the determination of the rate constant, k, (dimension: s^-1^, k) of hydrogen peroxide decomposition. The rate constant of the enzyme was determined by measuring the reduction in absorbance at 240 nm per minute. One unit (U) was defined as the amount of enzyme which decomposes 1 mol of H_2_O_2_ per min at 25 °C and pH 7.0 (35, 36).

*Statistical analysis*

Data were expressed as mean ± SEM. Statistical analyses were done using one-way ANOVA followed by Tukey’s *post hoc* test by SPSS 16. Statistical significance was defined as *p < *0.05.

## Results

*Fasting blood glucose*

**Figure 1:**
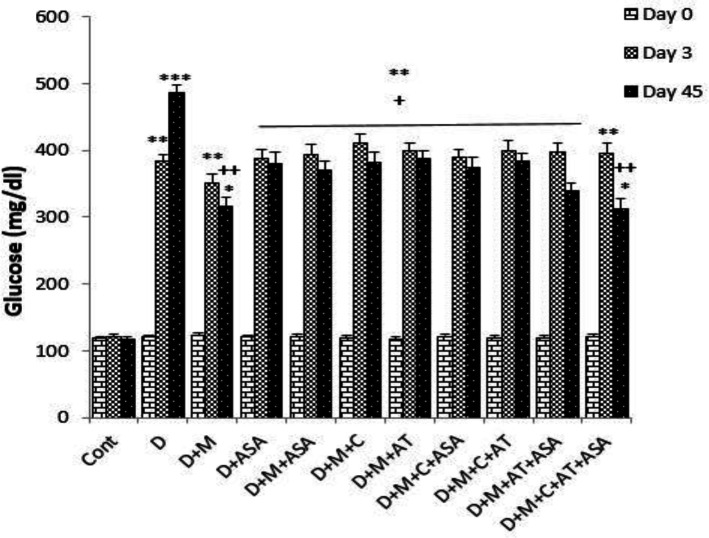
Fasting blood glucose levels on days 0, 3, and 45 of the experimental period. Data are shown as mean ± SEM. ^*^p < 0.05, ^**^p < 0.01 and ^***^p < 0.001 show significant differences as compared to control group. ^+^*p < *0.05 and ^++^*p < *0.01 show significant differences as compared to diabetic group (n = 8 in each group).

Fasting serum glucose levels on days 3 and 45 in the non-treated diabetic group (*i.e., *group D) and in groups that received different combinations of metformin, aspirin, atorvastatin, and captopril, were significantly higher than that of the control group (*p < *0.001 for the non-treated diabetic group on day 45, *p < *0.01 for the non-treated diabetic group on day 3 and also for groups that received different combinations of drugs on days 3 and 45, *p < *0.05 for D + M and D + M + C + AT + ASA groups on day 45). On day 45, fasting serum glucose levels in all drug-treated groups were significantly lower than that of the diabetic group (*p < *0.01 for D + M + C + AT + ASA and D + M groups and *p < *0.05 for other drug-treated groups, as compared to the diabetic group). There was no significant difference in serum glucose levels between the groups that received different combinations of these four drugs (Figure 1).

*Serum lipid profile*

**Figure 2:**
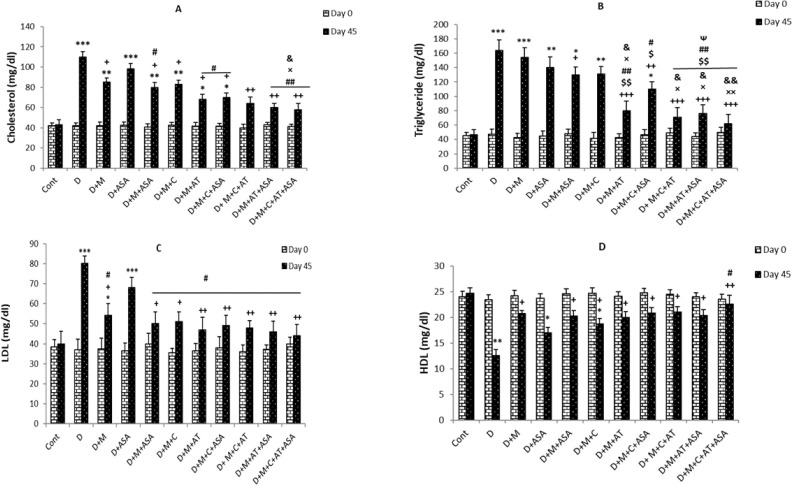
(A) Serum levels of cholesterol, (B) triglyceride, (C) LDL- cholesterol and (D) HDL- cholesterol. Data are shown as mean ± SEM. ^*^p < 0.05, ^**^p < 0.01 and ^***^p < 0.001 show significant differences as compared to control group; ^+^*p < *0.05, ^++^*p < *0.01, and ^+++^*p < *0.001 show significant differences as compared to diabetic group; ^$^*p < *0.05 and ^$$^*p < *0.01 show significant differences as compared to D + M group; ^#^*p < *0.05 and ^##^*p < *0.01 show significant differences as compared to D + ASA group; ^×^*p < *0.05 and ^××^*p < *0.01 show significant differences as compared to D + M + ASA group; ^&^*p < *0.05 and ^&&^*p < *0.01 show significant differences as compared to D + M + C group; and ^Ѱ^*p < *0.05 shows significant differences as compared to D + M + C + ASA group (n = 8 in each group).

On day 45, serum cholesterol levels in the non-treated diabetic group were significantly higher than the control group (*p < *0.001). All drug-treated groups, except the aspirin-only-treated group, showed significant decreases in serum cholesterol levels compared to the non-treated diabetic group (*p < *0.05 for groups that received one or two drugs but *p < *0.01 for groups that received combinations of three or four drugs). Also, cholesterol levels in groups that received combinations of three or four drugs (*i.e.,* D + M + C + ASA, D + M + C + AT, D + M + AT + ASA, and D + M + C + AT + ASA groups) showed significant decreases compared to groups that received one or two drugs (*p < *0.05 and *p < *0.01 compared to ASA group, *p < *0.05 compared to D + M + ASA group and *p < *0.05 compared to D + M + C group). Among the three groups that received combinations of two drugs, significant decreases in serum cholesterol levels were only observed in the D + M + ASA and D + M + AT groups, compared to the D + ASA group (Figure 2A).

The results showed significant increases in serum triglyceride levels in the non-treated diabetic group compared to the control group on day 45 (*p < *0.001); however, serum triglyceride levels in groups that received combinations of three or four drugs were significantly lower than those of non-treated diabetic group (*p < *0.01 to *p < *0.001). Among the three groups treated with combinations of two drugs, only the D+M+AT group showed significant decreases in serum triglyceride levels compared to the non-treated diabetic group (*p < *0.001). Also, triglyceride levels in groups that received combinations of three or four drugs (*i.e.,* D + M + C + ASA, D + M + C + AT, D + M + AT + ASA, and D + M + C + AT + ASA groups) and D + M + AT group were significantly reduced as compared to groups treated with one medication or combinations of two drugs (*p < *0.05 and *p < *0.01 compared to D + M, D + ASA, D + M + ASA and D + M + C groups) In D + M + C + AT, D + M + AT + ASA, and D + M + C + AT + ASA groups, there were significant decreases in serum triglyceride levels compared to the D + M + C + ASA group (Figure 2B).

On day 45, serum LDL- cholesterol levels were significantly higher in non-treated diabetic (*p < *0.001) and D+M groups (*p < *0.05) compared to the control group. All drug-treated groups, except the aspirin-only-treated group, showed significant decreases in serum LDL- cholesterol levels compared to the non-treated diabetic group (*p < *0.05 for groups that received one or two drugs and *p < *0.01 for groups that received combinations of three or four drugs and D + M + AT group compared to the diabetic group). Also, LDL- cholesterol levels in all groups treated with different combinations of drugs were significantly lower compared to the ASA-only-treated group (*p < *0.05) (Figure 2C).

Serum HDL- cholesterol levels on day 45, were significantly lower in non-treated diabetic (*p < *0.01), D + ASA (*p < *0.05) and D + M + C groups (*p < *0.05) compared to the control group. All drug-treated groups, except the aspirin-only-treated group, showed significant increases in serum HDL- cholesterol levels compared to the non-treated diabetic group (*p < *0.01 for the group that received a combination of four drugs and *p < *0.05 for other drug-treated groups compared to the non-treated diabetic group). Also, HDL- cholesterol levels showed significant increases in the group who received a combination of four drugs (D + M + C + AT + ASA group) compared to the ASA-only-treated group (*p < *0.05) (Figure 2D).

*Atherogenic index of plasma*

**Figure 3:**
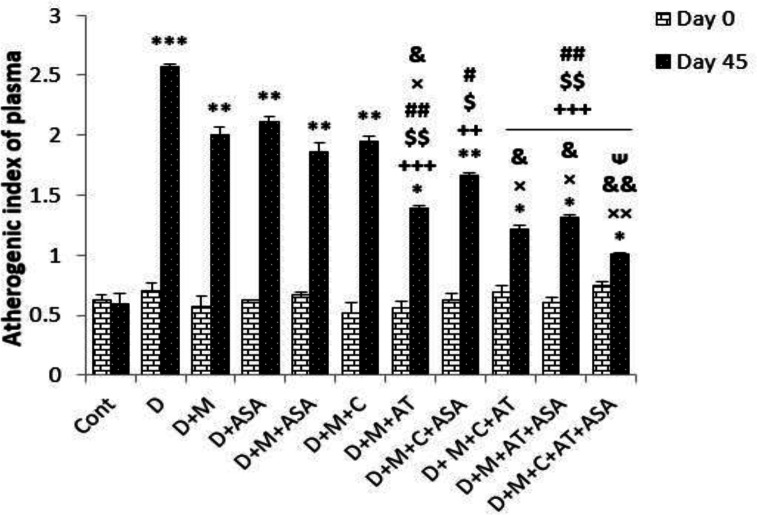
Atherogenic index of plasma (AIP). Data are shown as mean ± SEM. ^*^p < 0.05, ^**^p < 0.01, and ^***^p < 0.001 show significant differences as compared to control group; ^++^*p < *0.01, and ^+++^*p < *0.001 show significant differences as compared to diabetic group; ^#^*p < *0.05 and ^##^*p < *0.01 show significant differences as compared to D + ASA group; ^×^*p < *0.05 and ^××^*p < *0.01 show significant differences as compared to D + M + ASA group; ^&^*p < *0.05 and ^&&^*p < *0.01 show significant differences as compared to D + M + C group; ^$^*p < *0.05 and ^$$^*p < *0.01 show significant differences as compared to D + M group; and ^ᴪ^*p < *0.05 shows significant differences as compared to D + M + C + ASA group (n = 8 in each group)

Atherogenic index of plasma (AIP) in the non-treated diabetic group and in all groups that received different combinations of metformin, aspirin, atorvastatin, and captopril, was significantly higher than that of the control group (*p < *0.001 for the non-treated diabetic group, *p < *0.01 for all groups that received combinations of two or one drugs and D + M + C + ASA group, *p < *0.05 for groups that received combinations of three or four drugs and D + M + AT group) compared to the control group on day 45 (*p < *0.001); however, groups that received combinations of three or four drugs showed significantly lower AIP compared to non-treated diabetic group (*p < *0.01 to *p < *0.001). Among groups that received combinations of two drugs, only the D + M + AT group had significantly reduced AIP compared to the non-treated diabetic group. Also, groups that received combinations of three or four drugs (*i.e., *D + M + C + ASA, D + M + C + AT, D + M + AT +ASA, and D + M + C + AT + ASA groups) and D + M + AT group had significantly decreased AIP compared to groups that received one or combinations of two drugs (Figure 3).

*Malondialdehyde levels*

**Figure 4:**
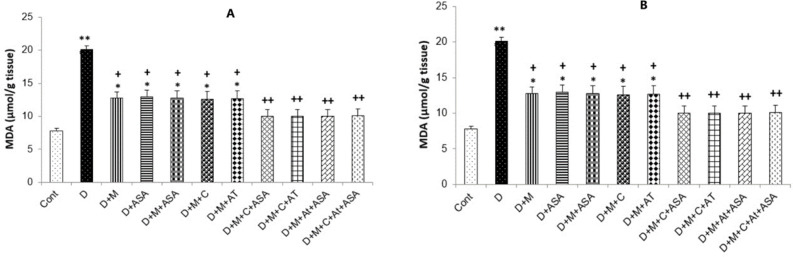
MDA concentration in the (A) heart and (B) kidney tissues. Data are shown as mean ± SEM. ^*^p < 0.05, ^**^p < 0.01 and ^***^p < 0.001 show significant differences as compared to control group and ^+^*p < *0.05 and ^++^*p < *0.01 show significant differences as compared to diabetic group (n = 8 in each group).

Malondialdehyde (MDA) levels in the heart and kidney tissues were collected from the non-treated diabetic group (*p < *0.001 and *p < *0.01, respectively) and in groups that received one or combinations of two drugs (*p < *0.05), were significantly higher than those of the control group. All drug-treated groups showed significantly lower MDA levels compared to the non-treated diabetic group (*p < *0.05 for groups that received one or combinations of two drugs and *p < *0.01 for groups that received combinations of three or four drugs) (Figure 4A and 4B).

*Total thiol concentrations*

**Figure 5.**
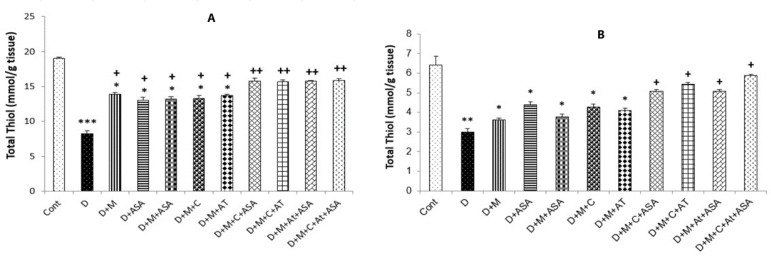
Total thiol concentrations in the (A) heart and (B) kidney tissues. Data are shown as mean ± SEM. ^*^p < 0.05, ^**^p < 0.01 and ^***^p < 0.001 show significant differences as compared to the control group. ^+^*p < *0.05 and ^++^*p < *0.01 show significant differences as compared to the non-treated diabetic group (n = 8 in each group)

Total thiol concentrations in the heart and kidney tissues collected from the non-treated diabetic group (*p < *0.001 and *p < *0.01, respectively) and in groups that received one or combinations of two drugs (*p < *0.05) were significantly lower than those of the control group. Total thiol concentrations in the heart tissue of all drug-treated groups were significantly higher compared to the non-treated diabetic group (*p < *0.05 for groups that received one or combinations of two drugs and *p < *0.01 for groups that received combinations of three or four drugs). In the kidney tissue, only in groups that received combinations of three or four drugs, significantly lower levels of total thiol were observed as compared to the non-treated diabetic group (*p < *0.05) (Figures 5A and 5B).

*SOD and CAT activities*

The SOD and CAT activities in the heart and kidney tissues of the non-treated diabetic group were significantly lower compared to the control group (*p < *0.001 and *p < *0.01, respectively). The heart tissues collected from all drug-treated groups had incredibly high levels of SOD activity compared to the non-treated diabetic group (*p < *0.05 for groups that received one drug or combinations of two medications and *p < *0.01 for groups that received combinations of three or four medications as compared to the diabetic group). In the kidney tissue obtained from groups that received combinations of three or four drugs and the D + M + C group, SOD activity was significantly increased compared to the non-treated diabetic group (*p < *0.05 for D + M + C + AT + ASA group and *p < *0.05 for other groups as compared to the diabetic group).

**Figure 6.**
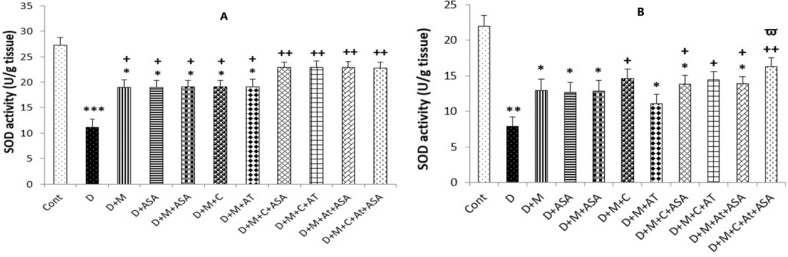
SOD activity in the (A) heart and (B) kidney tissues. Data are shown as mean ± SEM. ^*^p < 0.05, ^**^p < 0.01, and ^***^p < 0.001 show significant differences as compared to the control group; ^+^*p < *0.05, and ^++^*p < *0.01 show significant differences as compared to the diabetic group; and ^Ѱ^*p < *0.05 shows significant differences as compared to the D + M + AT treated group (n = 8 in each group).

**Figure 7:**
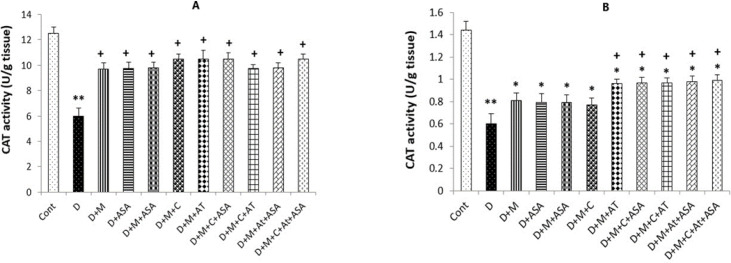
The CAT activity in (A) heart and kidney (B) tissues. Data were shown as mean ± SEM. ^*^p < 0.05 and ^**^p < 0.01 show significant differences as compared to the control group and ^+^*p < *0.05 shows significant differences as compared to the diabetic group (n = 8 in each group).

Also, significantly higher CAT activity was determined in the heart tissue collected from all drug-treated groups compared to the non-treated diabetic group (*p < *0.05); however, only kidney tissues collected from groups that received combinations of three or four medications presented significant increases in CAT activity compared to the diabetic group (*p < *0.05) (Figures 6A, 6B, 7A and 7B).

## Discussion

The current findings suggested that treatment with aspirin, atorvastatin, metformin, captopril and different combinations can rectify the imbalance between the generation of ROS and the scavenging enzyme activity in the heart and kidney tissues of diabetic animals. Consistent with our findings, several studies showed the antioxidant effect of aspirin, atorvastatin, metformin and ACE inhibitors that led to reduction of lipid peroxidation and increment of total thiol concentration, and SOD and CAT activities that can be favorable for the kidney and cardiovascular system (37-40).

In this study, more marked improvements of oxidative stress factors were observed in groups that received combinations of three or four medications compared to groups that received one drug or combinations of two drugs, indicating that co-administration of these drugs synergistically enhance their antioxidant effect. Synergistic effects of co-administration of these drugs may be mediated via various antioxidative mechanisms induced by each of these medications; thus, a combination of these agents presents enhanced antioxidant effects. In this regard, metformin can decrease ROS through various mechanisms including inhibition of complex I of the electron transport chain, activation of glucose-induced protein kinase C-β2, reduction of NAD(P)H oxidase activity, inducing thioredoxin expression through activation of the AMPK-FOXO3 (Activated protein kinase- Forkhead box O3) pathway and increasing the activity of the antioxidative system (41-44, 6). Aspirin can prevent the generation of oxyradicals through inhibition of prostaglandins synthesis and induction of heme oxygenase-1 (HO-1) expression (45, 46). In addition, aspirin can increase the activity of the antioxidative system (21). Antioxidant effect of atorvastatin in diabetes may be mediated through inhibition of Rac-1 and geranylgeranyl pyrophosphate (GGPP), induction of heme oxygenase, interference with NAD(P)H oxidase expression and activity and antagonization of the pro-oxidant effect of angiotensin II (47-49, 15). Finally, captopril can decrease ROS levels via suppressing angiotensin II production, which stimulates the production of superoxide radicals by NADPH oxidase and uncoupled nitric oxide (NO) synthase (50).

In this study, blood glucose, cholesterol, TG, and LDL levels and AIP decreased, but HDL levels increased in drug-treated groups after 45 days. It should be noted that the administration of a combination of these medications produced more marked beneficial effects than the administration of each drug alone. In the aspirin-only-treated group (D + ASA), no significant alterations in cholesterol, LDL- cholesterol and HDL- cholesterol levels were found as compared to the non-treated diabetic group while in groups treated with metformin (D + M) or groups that received combinations of metformin and captopril (D + M + C), atorvastatin (D + M + AT) or aspirin (D + M + ASA), these factors were significantly improved; therefore, it can be concluded that aspirin alone has no positive effect on the improvement of lipid profile but in combination with other drugs, it could significantly improve lipid profile. Recent studies also reported that high-dose aspirin influences lipid metabolism (23, 24). In the present study, metformin could improve lipid profile when administered alone or in combination with captopril. These results are in line with data reported by previous studies demonstrating that metformin, as an antihyperglycaemic drug, as well as captopril can improve glucose and lipid metabolism (51-53). 

In the present experiment, a comparison of the groups that received combinations of two medications indicated that a combination of metformin and atorvastatin (D + M + AT) could reduce cholesterol and LDL-cholesterol to a greater extent compared to the combination of metformin and aspirin (D + M + ASA) or captopril (D + M + C). Also, the combination of metformin and atorvastatin (D + M + AT) more markedly reduced TG than groups that received a combination of metformin, captopril and aspirin (D + M + C + ASA). Comparison of the groups that received combinations of three medications demonstrated that in groups where atorvastatin was present (*i.e., *D + M + C + AT and D + M + ASA + AT groups), there was a significant reduction in TG levels compared to the treatment that lacked atorvastatin (*i.e., *D + M + C + ASA group). These findings indicated the high capacity of atorvastatin in improving lipid profiles. In line with many previous studies, these results showed that atorvastatin has strong antihyperlipidemic activity (54).

Statins such as atorvastatin inhibit intracellular cholesterol storage by inhibiting HMG-COA reductase. As a result, they increase the expression of LDL receptors. Increased expression of these receptors led to increases in blood LDL clearance rate and decrease in plasma cholesterol levels (22).

The role of AMPK in the lipid metabolism induced by metformin and aspirin was reported (55). Metformin and aspirin suppress ATP utilization pathways such as lipid synthesis by activating AMPK. Metformin also reduces the concentration of cholesterol and LDL by inhibiting the intestinal absorption of bile acids (55). Moreover, captopril reduces insulin-stimulated lipogenesis by reducing glycerol synthesis from glucose. In other words, captopril reduces the lipogenesis capacity of adipocytes (56). 

In this study, there was a significant decrease in AIP in groups that received combinations of three or four drugs compared to the non-treated diabetic group, but there was no significant reduction in AIP in groups treated with one or two drugs. One of the main risk factors and prognosticator for CVD (cardiovascular disease) is plasma lipid profile. AIP represents the correct relation between atherogenic and protective lipoprotein. Serious increase in AIP was observed as reflected by augmented TC, TG and LDL-C and reduced HDL-C. AIP act as a prognosticator for atherosclerosis, and can be used as an accurate indicator for cardiovascular risk factors (20, 21). Given that increasing evidence suggests AIP as an indicator of atherosclerosis, these results suggest that combinations of aspirin, atorvastatin, metformin and captopril could reduce AIP and consequently decrease CVD risk factors in DM (28). Moreover, comparing the groups that received combinations of two drugs showed that only in the D + M + AT group, AIP was significantly decreased compared to the non-treated diabetic group. This shows the high capacity of atorvastatin to improve lipid profile and, therefore, reduce AIP and CVD risk factors. 

According to these results, to improve the lipid profile, the efficacy of different combinations was in the following order: a combination of four drugs > combinations of three drugs > combination of two drugs > aspirin alone; thus, it can be concluded that these drugs probably produce synergistic effects in terms of improvement of lipid profiles when administered together.

Very few studies were conducted to evaluate the effects of different combinations of these medications and there is little information in this regard. Berardis *et al.* showed putative additive effects of co-administration of aspirin and statins to control cardiovascular risk factors in diabetes (57). It was also revealed that the combination of statins with angiotensin II system inhibitors or AT-1 receptors blockers had more marked effects on oxidative stress (58, 59). Tousoulis showed that co-treatment with metformin and atorvastatin could be regarded as a useful intervention with significant antioxidant and antihyperlipidemic activities as reflected by reduced lipid peroxidation in diabetic patients (60).

The current findings suggested that in diabetic rats, oxidative stress was induced; however, high-dose aspirin in combination with metformin, captopril and atorvastatin can exert synergism in terms of rectifying the imbalance between ROS generation and scavenging enzymes activity and improving diabetic hyperglycemia and hyperlipidemia.

## Conclusion

This study indicated that combinations of captopril, aspirin, atorvastatin and metformin could improve glucose and lipid metabolism and oxidative stress markers in the kidneys and heart tissues of diabetic rats to a greater extent as compared to their single administration.
